# Supplementary material for: Evaluation of accuracy, exclusivity, limit-of-detection and ease-of-use of LumiraDx™: An antigen-detecting point-of-care device for SARS-CoV-2
Source: Infection. 2021 Aug 12;50(2):395–406. doi: 10.1007/s15010-021-01681-y (PMC8358901; doi:10.1007/s15010-021-01681-y)
Supplement: Supplementary file 1 — Supplementary file1 (DOCX 1599 KB) [file 15010_2021_1681_MOESM1_ESM.docx]

**Supplementary material**

**Table of content**

[**(A)** **Table 1: Study Team** 2](#_Toc77241288)

[**(B)** **Section: Questionnaire for study participants** 3](#_Toc77241289)

[**(C)** **Section: System Usability Scale (SUS)** 6](#_Toc77241290)

[**(D)** **Section: Ease-of-Use Assessment (EoU)** 7](#_Toc77241291)

[**(E)** **Figure 1: Matrix for Ease-of-Use Assessment** 18](#_Toc77241292)

[**(F)** **Table 2: Detailed list of symptoms for all PCR positives** 20](#_Toc77241293)

[**(G) Table 3: Antigen-based RDT with test result, CT values and viral load for PCR positive participants in Berlin and Heidelberg** 25](#_Toc77241294)

[**(H) Figure 2: Correlation between the cut-off-index value and the viral load for all RT-PCR positive cases** 34](#_Toc77241295)

[**(I) Table 4: Discrepant analysis** 35](#_Toc77241296)

[**(J) Figure 3: Specific infectivity of virus stocks** 37](#_Toc77241297)

[**(K) Table 5: Exclusivity testing of LumiraDx™** 38](#_Toc77241298)

[**(L) Figure 4: System Usability Score and Ease-of-Use assessment results** 39](#_Toc77241299)

[**(M) Table 6: Comorbidities and list of symptoms of participants overall, Berlin and Heidelberg** 40](#_Toc77241300)

[**(N) Table 7: Sensitivity and Specificity overall and by subgroups** 41](#_Toc77241301)

# **Table 1: Study Team**

| Department of Public Health Rhein Neckar Region, Heidelberg, Germany | Dr. K. Assaad, |
| --- | --- |
|  | Dr. A. Fuhs |
|  | C. Harter |
|  | C. Schulze |
|  | G. Schmitt |
| Division of Clinical Tropical Medicine, Heidelberg University Hospital, Germany | Anja Klemmer |
|  | Rico Müller |
|  | Martina Fink |
|  | Mathilde Fougereau |
|  | Maximilian Schirmer |
|  | Annika Small |
|  | Matthias Meinlschmidt |
|  | Valerie Dürr |
|  | Alina Schuckert |
|  | Ann-Kathrin Backes |
|  | Salome Steinke |
|  | Henrik Ellinghaus |
|  | Loai Abutaima |
| Institute of Tropical Medicine and International Health, Charité – Universitätsmedizin Berlin, Berlin, Germany | Mandy Kollatzsch |
|  | Mia Wintel |
|  | Franka Kausch |
|  | Franziska Hommes |
|  | Alisa Bölke |
|  | Julian Bernhard |
|  | Claudia Hülso |
|  | Elisabeth Linzbach |
| Medical Directorate, Charité – Universitätsmedizin Berlin, Berlin, Germany | Heike Rössig |
| Institute of Tropical Medicine and International Health, Charité – Universitätsmedizin Berlin, Berlin, Germany | Maximilian Gertler |
| Charité Comprehensive Cancer Center, Charité – Universitätsmedizin Berlin, Berlin, Germany | Susen Burock |
| Department of Pediatric Surgery, Charité – Universitätsmedizin Berlin, Berlin, Germany | Katja von dem Busche |
| Berlin Institute for Clinical Teratology and Drug Risk Assessment in Pregnancy, Institute of Clinical Pharmacology and Toxicology, Charité – Universitätsmedizin Berlin, Berlin, Germany | Stephanie Patberg |

#

# **Section: Questionnaire for study participants**

**We invite you to participate in this survey. The survey serves to understand the diagnostic process and the disease and factors related to SARS-CoV-2 (novel coronavirus) infection.**

**Your answers will be kept strictly confidential and will not have a negative impact on your care. Participation in the study is voluntary and you have the option to skip questions that you do not want to answer.**

**The survey is expected to take 15-20 minutes. Thank you for your understanding and cooperation!**

| Postal code | *(Free text)* |
| --- | --- |
| Gender | - Male - Female - Diverse |
| How tall are you (in centimetres)? | *(Free text)* |
| How much do you weight (in kilograms)? | *(Free text)* |

**Symptoms that you attribute to the possible COVID-19**

| Did you have any symptoms of possible COVID-19 on the day of the test? | - No - Yes |
| --- | --- |
| Increased temperature / fever? | - No - Yes |
| Did you measure your fever? | - No - Yes |
| Highest temperature (in Celsius) | *(Free text)* |
| Cough | - No - Yes |
| Do you have a productive cough? | - No - Yes |
| Sore throat | - No - Yes |
| Shortness of breath | - No - Yes |
| Muscle pain / Body aches | - No - Yes |
| Fatigue | - No - Yes |
| Headache | - No - Yes |
| Runny nose | - No - Yes |
| Chest pain | - No - Yes |
| Diarrhea | - No - Yes |
| Nausea / vomiting | - No - Yes |
| Loss of taste or smell | - No - Yes |
| Other | - No - Yes |
| If yes, please specify | *(Free text)* |
| The earliest onset of symptoms attributed to possible COVID-19 | *(Day / Month / Year)* |
| How sick did you feel on the day of the test? | - Normal unrestricted activity as before the illness - Restriction with physical exertion, but able to walk; light physical work or work while sitting, e.g., light housework or office work, possible - Able to walk, self-sufficiency possible, but not able to work; can get up more than 50% of the waking time - Only limited self-sufficiency possible; 50% or more of the waking time tied to bed or chair - Completely in the need of care, no self-sufficiency possible; completely tied to bed or chair |
| Did you previously test negative within the last 10 days? | - No - Yes |
| If yes, when | *(Day / Month / Year)* |
| If yes, where | - University clinic – inpatient - University clinic – outpatient - Drive-in - Other |
| Other, please specify | *(Free text)* |
| Do you know where you might have been infected with COVID-19? | - Household contact - Social contact - Work contact - Contact in university / school / kindergarten from you or a child in the family - Travel to risk area - Do not know - Other |
| Risk area | *(Free text)* |
| Other: please describe | *(Free text)* |

**Do you have any pre-existing conditions?**

| Which of the following lung disease(s) do you have? | - Asthma - Chronic Obstructive Pulmonary Disease – COPD - Breathing disorders during sleep - Obstructive Sleep Apnea – OSAS - Interstitial Lung Disease - Lung Cancer - Other - None |
| --- | --- |
| What other lung diseases do you have? | *(Free text)* |
| Cardiovascular diseases (e.g. hypertension, stroke, etc.) | - No - Yes |
| Chronic kidney disease | - No - Yes |
| Diabetes | - No - Yes |
| Autoimmune Disease (e.g. Rheumatoid Arthritis, MS) | - No - Yes |
| HIV | - No - Yes |
| Overweight | - No - Yes |
| Other, please specify | *(Free text)* |

# **Section: System Usability Scale (SUS)**

"Evaluation of the performance of novel rapid diagnostics for SARS-CoV-2 at point-of-care"

***System Usability Scale (SUS)***

© Digital Equipment Corporation, 1986 adapted format, version 1.0: 02/05/20

Name of the test: _______________________________________________________________


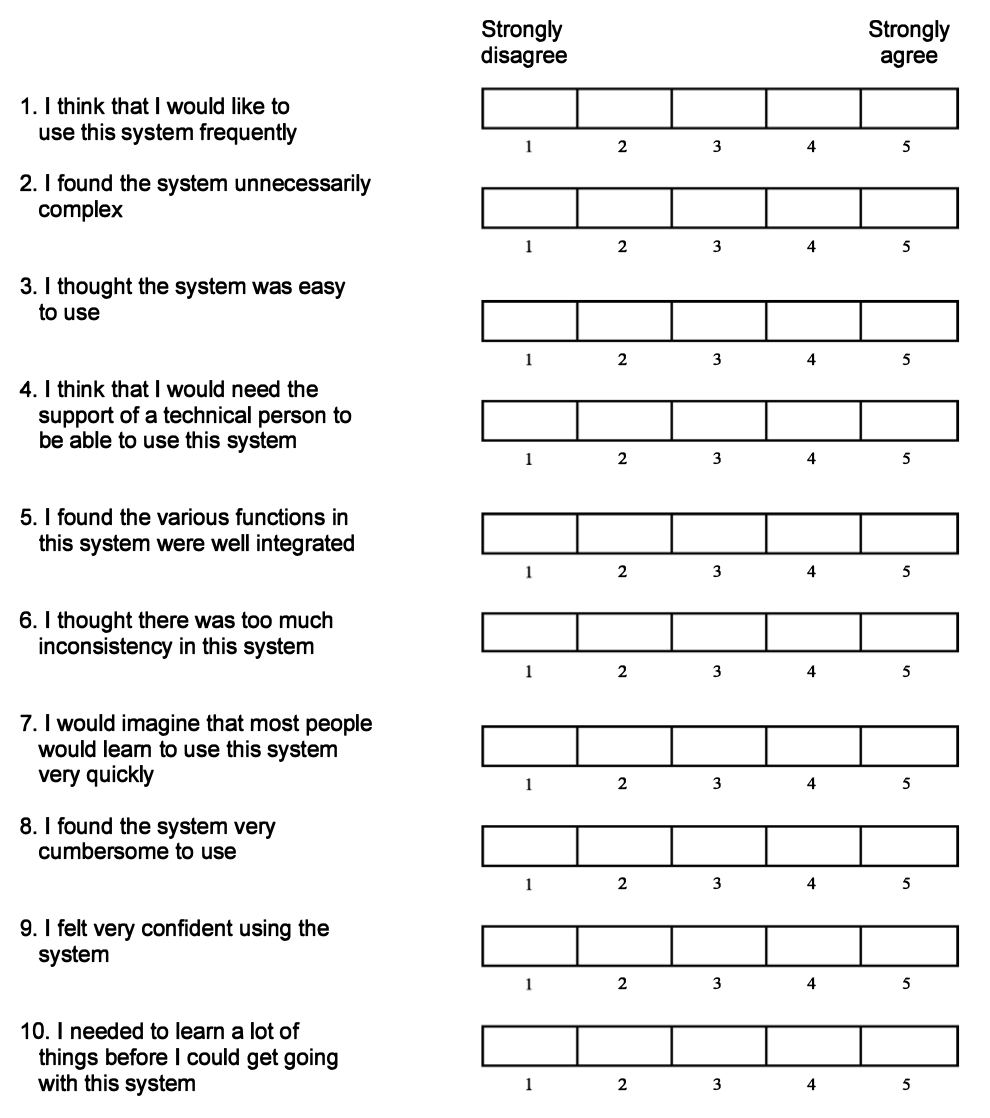
User identifier and study site: _________________________ Date: _______________________

***Using SUS***

The SU scale is generally used after the respondent has had an opportunity to use the system being evaluated, but before any debriefing or discussion takes place. Respondents should be asked to record their immediate response to each item, rather than thinking about items for a long time.

All items should be checked. lf a respondent feels that they cannot respond to a particular item, they should mark the center point of the scale.

# **Section: Ease-of-Use Assessment (EoU)**

"Evaluation of the performance of novel rapid diagnostics for SARS-CoV-2 at point-of-care"

Thank you for your time to answer this questionnaire (about 20 minutes).

*Your input is very valuable!*

***OVERALL QUESTIONS***

1. **User identifier**
2. **Date of filling the questionnaire**
3. **In which country do you currently work?**
4. **At which facility / study site do you currently work?**

*Mark only one oval.*

|  | Reilingen (Heidelberg) |
| --- | --- |
|  | Berlin |
|  | Liverpool |
|  | Macae CTC |
|  | Marica (Guapi) |
|  | UFRJ |

1. **Which test are you assessing?**

*Mark only one oval.*

|  | Coris Bioconcept Respi Strip |
| --- | --- |
|  | Bioeasy FIA |
|  | Bioeasy Colloidal Gold |
|  | SD Biosensor Standard F (Flourescence) |
|  | SD Biosensor Standard Q |
|  | Rapigen Biocredit Colloidal Gold |
|  | Abbott Panbio |
|  | LumiraDx |

1. **Approximately how many times did you perform this test?**

*Mark only one oval.*

|  | Only observed use |
| --- | --- |
|  | < 10 |
|  | 10 – 100 |
|  | > 100 |

1. **Approximately how many times did you observe the use of this test (not performed yourself)?**

*Mark only one oval.*

|  | < 10 |
| --- | --- |
|  | 10 – 50 |
|  | 50 – 100 |
|  | > 100 |

1. **What is your profession?**

****

1. **How many years of laboratory experience do you have?**

** **

1. **How many years of working experience in limited resource settings do you have?**

** **

1. **How much experience do you have with interpreting the results of lateral flow tests or rapid diagnostics (e.g. for HIV, malaria, pregnancy)?**

Please note that we refer here to your experience with INTERPRETING the test results. If you do not conduct the test yourself, but do inform patients about the test results, we also consider that as experience with INTERPRETING the test results.

*Mark only one oval.*

|  | None |
| --- | --- |
|  | < 1 year |
|  | 1 – 3 years |
|  | > 3 years |

***TEST SPECIFIC QUESTIONS***

*TRAINING*

1. **How satisfied were you with the following components of the test training?**

*Mark only one oval per row.*

|  | Very satisfied | Satisfied | Neither | Dissatisfied | Very dissatisfied |
| --- | --- | --- | --- | --- | --- |
| Instructions for Use |  |  |  |  |  |
| Standard Operating Procedures |  |  |  |  |  |
| Face to face demonstration |  |  |  |  |  |

1. **What additional materials (if any) do you think should be provided as part of the training?**

|  | None |
| --- | --- |
|  | Other: _______________________________________________________________________________ |

1. **How long should be the training of this test?**

*Mark only one oval.*

|  | Self-explanatory, no need for training |
| --- | --- |
|  | 1 - 2 hours |
|  | 2 - 4 hours |
|  | Half a day |
|  | Full day |

1. **Do you consider proficiency testing necessary?**

Proficiency testing as in assessing the user's performance or ability to run the test following the training.

*Mark only one oval.*

|  | Yes |
| --- | --- |
|  | No |
|  | Other: : ______________________________________________________________________________ |

1. **Please comment here on the need for proficiency testing**

___________________________________________________________________________________________

1. **Do you think that a company training is necessary?**

|  | Yes |
| --- | --- |
|  | No |
|  | Other: : _____________________________________________________________________________ |

1. **Please comment on having the company come to do an in-person training.**

__________________________________________________________________________________________

1. **After how many of these tests do you feel you could perform the test on your own (having access to the training material)?**

*Mark only one oval.*

|  | 1 – 2 tests |
| --- | --- |
|  | 3 – 5 tests |
|  | 6 – 10 tests |
|  | > 10 tests |

*ASSESSMENT OF TEST COMPONENTS*

1. **How satisfied are you with the quality of each of the components in the Test Strip Carton (in terms of ease of use and fit for purpose)?**

*Mark only one oval per row*

|  | Very satisfied | Satisfied | Neither | Dissatisfied | Very dissatisfied |
| --- | --- | --- | --- | --- | --- |
| External paper box of kit |  |  |  |  |  |
| Extraction tube |  |  |  |  |  |
| Filter cap |  |  |  |  |  |
| Test cartridge / device |  |  |  |  |  |
| Test cartridge packing / pouch |  |  |  |  |  |

1. **Which kit component(s) should be improved in your opinion (if any)?**

*Please specify why and how*

___________________________________________________________________________________________­­

1. **Overall, how satisfied are you with the kit components?**

*Mark only one oval.*

|  | 1 | 2 | 3 | 4 | 5 |  |
| --- | --- | --- | --- | --- | --- | --- |
| Very satisfied |  |  |  |  |  | Very dissatisfied |

1. **How satisfied are you with the overall design of the test strip in terms of the following features?**

*Mark only one oval per row.*

|  | Very satisfied | Satisfied | Neither | Dissatisfied | Very dissatisfied |
| --- | --- | --- | --- | --- | --- |
| Size of test strip |  |  |  |  |  |
| Size of the well to add sample mix |  |  |  |  |  |

1. **How satisfied are you with the quality of the quality controls in terms of ease of use and fit for purpose?**

|  | 1 | 2 | 3 | 4 | 5 |  |
| --- | --- | --- | --- | --- | --- | --- |
| Very satisfied |  |  |  |  |  | Very dissatisfied |

1. **How useful do you find the inclusion of a positive quality control?**

|  | 1 | 2 | 3 | 4 | 5 |  |
| --- | --- | --- | --- | --- | --- | --- |
| Very useful |  |  |  |  |  | Not useful |

1. **How useful do you find the inclusion of a negative quality control?**

|  | 1 | 2 | 3 | 4 | 5 |  |
| --- | --- | --- | --- | --- | --- | --- |
| Very useful |  |  |  |  |  | Not useful |

1. **Please comment on the added value and quality of the quality controls.**

___________________________________________________________________________________________

1. **How satisfied are you with the quality of the reader in terms of its ease of use and fit for purpose?**

|  | 1 | 2 | 3 | 4 | 5 |  |
| --- | --- | --- | --- | --- | --- | --- |
| Very satisfied |  |  |  |  |  | Very dissatisfied |

1. **Overall, how satisfied are you with the reader?**

|  | 1 | 2 | 3 | 4 | 5 |  |
| --- | --- | --- | --- | --- | --- | --- |
| Very satisfied |  |  |  |  |  | Very dissatisfied |

1. **Which component(s) of the reader (if applicable) should be improved in your opinion (if any)? Please specify why and how.**

___________________________________________________________________________________________

*ASSESSMENT OF TEST*

1. **Please determine the difficulty of the following steps:**

Please consider your day-to-day/routine workload (or that of the people in the lab/area where this test could be implemented) to answer this question. Please leave any steps blank if not applicable.

*Mark only one oval per row.*

|  | Very easy | Easy | Neither | Difficult | Very difficult |
| --- | --- | --- | --- | --- | --- |
| a) Check expiry date |  |  |  |  |  |
| b) Label the extraction tube with patient identifier |  |  |  |  |  |
| c) Open the extraction tube by removing the seal |  |  |  |  |  |
| d) Insert the swab into the tube |  |  |  |  |  |
| e) Ease of swab extraction procedure |  |  |  |  |  |
| f) Ability to perform swab extraction procedure consistently |  |  |  |  |  |
| g) Ability to maintain cleanliness of ancillary devices (e.g. pipette) in order to avoid cross contamination |  |  |  |  |  |
| h) Remove the test cartridge from the pouch |  |  |  |  |  |
| i) Ease of transferring sample onto device |  |  |  |  |  |
| j) Ease of transferring exact quantity into the sample well |  |  |  |  |  |
| k) Ease of using instrument |  |  |  |  |  |
| l) Trouble shooting |  |  |  |  |  |

1. **How satisfied are you with the logical sequence of steps?**

*Mark only one oval.*

|  | 1 | 2 | 3 | 4 | 5 |  |
| --- | --- | --- | --- | --- | --- | --- |
| Very satisfied |  |  |  |  |  | Very dissatisfied |

1. **Overall, how difficult did you find the steps?**

*Mark only one oval.*

|  | 1 | 2 | 3 | 4 | 5 |  |
| --- | --- | --- | --- | --- | --- | --- |
| Very easy |  |  |  |  |  | Very difficult |

1. **In general, how satisfied are you with the time relevant components (ex. are the steps relatively short, are there many specific timed steps that make it hard to keep track)?**

*Mark only one oval.*

|  | 1 | 2 | 3 | 4 | 5 |  |
| --- | --- | --- | --- | --- | --- | --- |
| Very satisfied |  |  |  |  |  | Very dissatisfied |

1. **Please assess the time relevant components of the test.**

*Mark only one oval per row.*

|  | ≤ 2 min | 3 to 5 min | 6 to 10 min | > 10 min |
| --- | --- | --- | --- | --- |
| Pre analytic time (i.e. from when you get the swab to when you start incubation) |  |  |  |  |
| Incubation time (how long do you have to wait to get the results once you add your sample to the strip) |  |  |  |  |
| Analytic time (time needed to analyse the results) |  |  |  |  |

1. **In your opinion, about how many patients could be tested with this test in an 8-hour day (with one instrument and one sample method only)?**

*Mark only one oval per row.*

|  | < 10 |
| --- | --- |
|  | 10 – 50 |
|  | 50 – 100 |
|  | > 100 |

1. **Please comment here if you see any potential issues or room for improvement.**

___________________________________________________________________________________________

1. **How did you find the results read-out in the following areas?**

*Mark only one oval per row.*

|  | Very easy | Easy | Neither | Difficult | Very difficult |
| --- | --- | --- | --- | --- | --- |
| a) Read-out from Reader |  |  |  |  |  |
| b) Interpretation of the test result |  |  |  |  |  |

1. **Do you foresee any issues with reading these results considering the lighting conditions in the
    settings you currently work or have experience with?**

*Mark only one oval.*

|  | Yes (please explain below) |
| --- | --- |
|  | No |

**If yes, please explain here**

_______________________________________________________________________________________________

*OVERALL ASSESMMENT*

1. **Overall, how did you find the use of this rapid COVID-19 diagnostic tool:**

*Mark only one oval.*

|  | 1 | 2 | 3 | 4 | 5 |  |
| --- | --- | --- | --- | --- | --- | --- |
| Very easy |  |  |  |  |  | Very difficult |

1. **Please comment here on the use:**

___________________________________________________________________________________________

­­­­­­­­­­­­­­

1. **Which option(s) do you consider feasible in your setting?**

|  | Sequential testing (run tests one by one) ONLY |
| --- | --- |
|  | Sequential testing AND batch testing (run multiple tests at the same time) |

1. **Which aspect(s) of this test could cause difficulties in its day-to-day use?** *Tick all that apply.*

|  | Hands-on time |
| --- | --- |
|  | Tatal assay time to result |
|  | Batch processing |
|  | Throughput |
|  | Test results interpretation |
|  | Overall number of steps |
|  | Time sensitive steps |
|  | Cartridge design |
|  | Quality of material |
|  | Training requirements |
|  | Storage conditions and stability |
|  | Waste management requirements |
|  | I don’t know |
|  | None, I see no barriers for implementation |

1. **Please give a short explanation for each of the aspects you selected above e.g. what could be the
    challenges in the day-to-day use:**

_______________________________________________________________________________________________

*SETTINGS UF USE*

1. **Do you see this test being used in its current form in your setting in your country?**

*Mark only one oval.*

|  | Yes (please explain below) |
| --- | --- |
|  | No (please explain below) |
|  | I don’t know |

Please elaborate:

_______________________________________________________________________________________________

1. **If yes, at which health care level(s) do you see this test being implemented in your country**

*Tick all that apply.*

|  | Family doctor / General physician |
| --- | --- |
|  | Peripheral hospital / lab |
|  | Reference hospital / lab |
|  | At a testing site operated by traines staff without specific laboratory expertise |

1. **If you don't see this test being used in its current form, which aspects should be changed to make it suitable for use in your setting in your country:**

___________________________________________________________________________________________

**48. If you don't see this test being used in its current form, which aspects should be changed to make it suitable for use in your setting in low- and middle-income countries?**

_______________________________________________________________________________________________

**49. Anything else you would like to add?**

**_______________________________________________________________________________________________**

**THANK YOU VERY MUCH**

# **Figure 1: Matrix for Ease-of-Use Assessment**


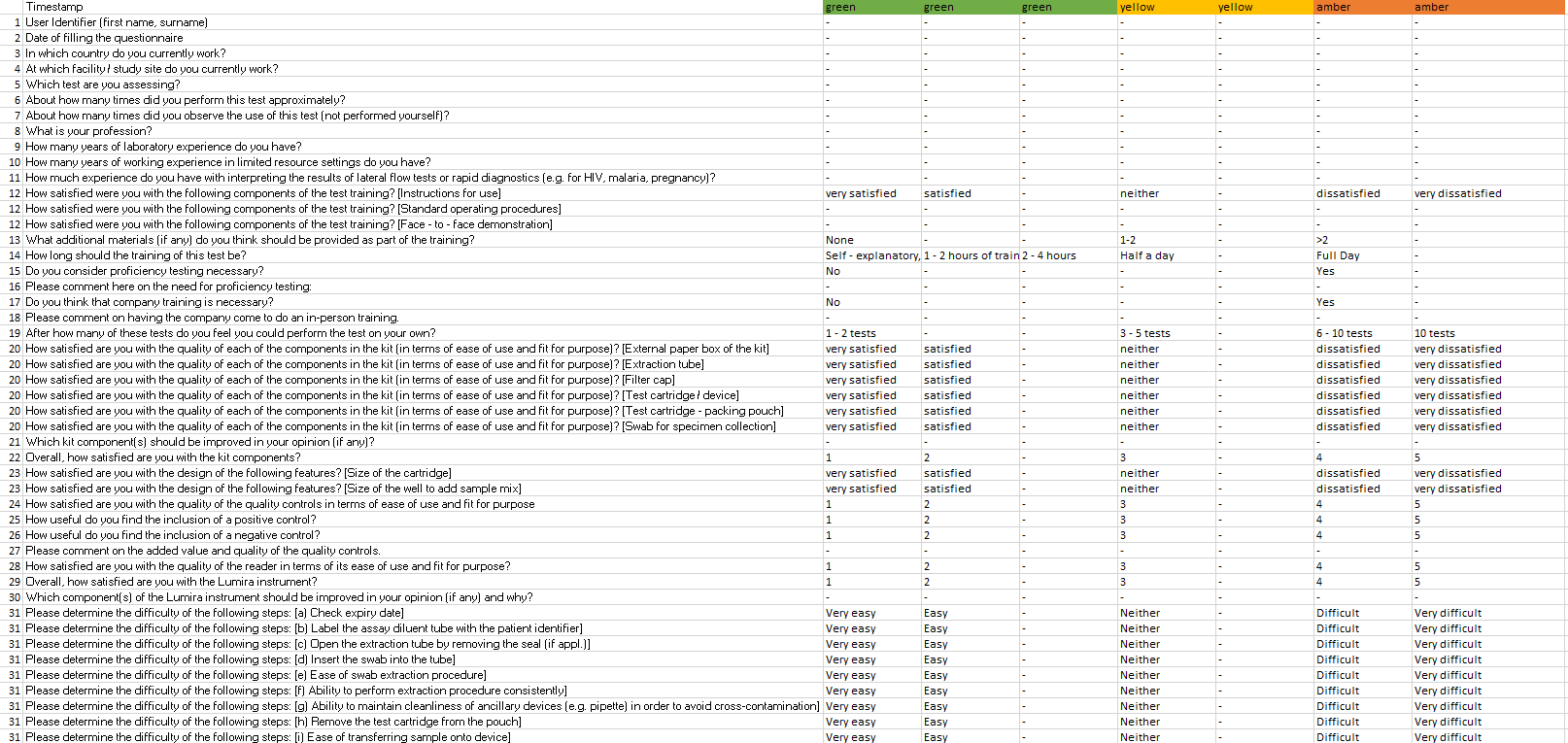


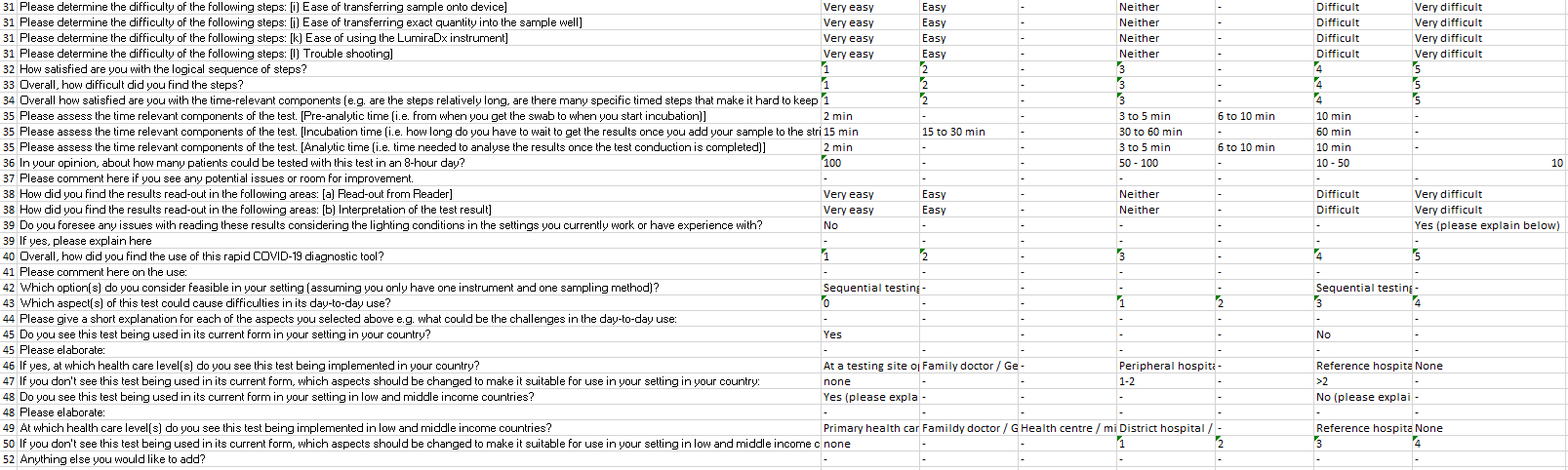


1. **Table 2: Detailed list of symptoms for all PCR positives**

| **Viral load**  ***(log_10_RNA copies/mL)*** | **Result Ag-RDT** | **Increased temperature/**  **fever** | **Cough** | **Do you have a productive cough?** | **Sore throat** | **Shortness of breath** | **Muscle pain/Body aches** | **Fatigue** | **Headache** | **Runny nose** | **Chest pain** | **Diarrhea** | **Nausea/**  **vomiting** | **Loss of taste /smell** | **Other symptoms** |
| --- | --- | --- | --- | --- | --- | --- | --- | --- | --- | --- | --- | --- | --- | --- | --- |
| 6.85 | positive | No | Yes | Yes | No | No | No | Yes | Yes | Yes | No | No | No | Yes |  |
| 7.56 | positive | No | Yes | No | Yes | No | Yes | Yes | Yes | Yes | No | No | No | Yes | No |
| 7.97 | positive | Yes | Yes | No | Yes | No | Yes | Yes | Yes | Yes | No | No | No | Yes | Yes |
| 7.76 | positive | No | No | No | Yes | Yes | Yes | Yes | Yes | No | Yes | No | No | No | No |
| 8.00 | positive | Yes | Yes | No | No | No | Yes | Yes | Yes | No | Yes | No | No | No | Yes |
| 7.61 | positive | Yes | Yes | Yes | No | No | Yes | Yes | No | No | No | No | No | No | No |
| 7.11 | negative | No | No | No | No | No | No | No | No | No | No | No | No | Yes | No |
| 8.15 | positive | Yes | Yes | No | Yes | No | Yes | No | Yes | Yes | No | No | No | Yes | No |
| 6.82 | positive |  |  |  |  |  |  |  |  |  |  |  |  |  |  |
| 5.99 | positive | Yes | No | No | No | No | Yes | Yes | No | No | No | No | No | No | No |
| 7.79 | positive | No | Yes | No | Yes | No | Yes | No | Yes | No | No | No | No | No | No |
| 3.99 | positive | No | No | No | No | Yes | No | Yes | Yes | No | No | No | No | No | No |
| 6.32 | positive | No | Yes | Yes | No | No | Yes | Yes | Yes | Yes | No | No | No | No | No |
| 8.38 | positive | No | Yes |  | Yes | No | Yes | Yes | Yes | Yes | No | No | No | No | No |
| 4.26 | negative | No | No | No | No | No | Yes | Yes | No | No | No | No | No | No | Yes |
| 7.73 | positive | No | No | No | No | No | Yes | Yes | Yes | No | No | No | No | Yes | Yes |
| 8.48 | positive | No | Yes | No | Yes | No | No | Yes | Yes | Yes | No | No | No | Yes | No |
| 8.20 | positive | Yes | Yes | No | No | Yes | Yes | Yes | Yes | Yes | No | Yes | No | Yes | No |
| 8.48 | positive | No | Yes | No | No | Yes | Yes | Yes | No | Yes | No | No | No | Yes | No |
| 8.32 | positive | Yes | Yes | Yes | No | No | Yes | No | Yes | Yes | No | No | No | No | No |
| 6.18 | positive | Yes | No | No | No | No | No | Yes | No | Yes | No | No | No | Yes | Yes |
| 3.78 | negative | No | Yes | No | Yes | No | No | No | No | Yes | No | No | No | Yes | No |
| 7.61 | positive |  |  |  |  |  |  |  |  |  |  |  |  |  |  |
| 5.56 | positive |  | Yes | No | No | No | Yes | Yes | Yes | Yes | No | No | No | Yes | No |
| 3.99 | positive |  |  |  |  |  |  |  |  |  |  |  |  |  |  |
| 7.73 | positive | Yes | Yes | No | Yes | No | Yes | Yes | Yes | No | Yes | Yes | No | No | No |
| 8.08 | positive | No | No | No | No | No | Yes | Yes | Yes | No | No | Yes | No | No | No |
| 7.53 | positive | Yes | Yes | No | Yes | No | Yes | Yes | Yes | Yes | No | Yes | No | Yes | No |
| 7.56 | positive | No | Yes | No | Yes | No | No | No | Yes | Yes | No | No | No | No | No |
| 7.83 | positive | Yes | No | No | No | No | Yes | Yes | Yes | No | No | No | No | No | No |
| 6.67 | positive |  |  |  |  |  |  |  |  |  |  |  |  |  |  |
| 6.58 | positive | No | Yes | No | No | No | Yes | Yes | Yes | No | Yes | Yes | No | No | No |
| 4.90 | positive | Yes | No | No | Yes | No | No | No | Yes | No | No | No | No | No | No |
| 5.40 | positive | No | Yes | No | Yes | No | No | Yes | Yes | No | No | No | Yes | No | No |
| 2.95 | negative |  |  |  |  |  |  |  |  |  |  |  |  |  |  |
| 8.59 | positive | Yes | Yes | Yes | No | No | Yes | No | No | Yes | No | No | No | No | No |
| 6.04 | positive | Yes |  |  |  |  |  |  |  |  |  |  |  |  |  |
| 4.81 | negative | No | Yes | No | No | No | No | Yes | Yes | No | No | No | No | Yes |  |
| 8.08 | positive | No | Yes | No | No | No | No | Yes | No | No | No | No | No | No | No |
| 7.41 | positive | Yes | No | No | No | No | Yes | Yes | Yes | No | No | Yes | No | No | No |
| 8.00 | positive | No | No | No | Yes | No | No | No | No | Yes | No | No | No | No | No |
| 4.79 | positive | Yes | Yes | Yes | No | No | Yes | Yes | Yes | No | No | Yes | No | No | No |
| 6.94 | positive | Yes | No | Yes | No | No | Yes | Yes | No | Yes | No | No | No | Yes |  |
| 5.52 | positive | No | No | No | No | No | Yes | Yes | Yes | No | No | Yes | No | No | No |
| 7.04 | positive | No | No | No | Yes | No | Yes | Yes | Yes | No | No | No | No | Yes | No |
| 6.28 | positive |  |  |  |  |  |  |  |  |  |  |  |  |  |  |
| 8.11 | positive | No | Yes | Yes | No | No | No | Yes | No | Yes | No | No | No | Yes | No |
| 5.11 | positive | Yes | Yes | Yes | Yes | No | Yes | Yes | Yes | Yes | Yes | No | No | Yes | No |
| 7.71 | positive | Yes | Yes | No | Yes | No | Yes | Yes | Yes | Yes | No | No | No | No | No |
| 3.66 | negative | Yes | Yes | No | Yes | No | No | Yes | Yes | No | No | Yes | No | Yes | Yes |
| 6.82 | positive |  |  |  |  |  |  |  |  |  |  |  |  |  |  |
| 7.15 | positive | No | No | No | No | No | No | No | No | Yes | No | No | No | No | No |
| 7.85 | positive | No | No | No | Yes | Yes | Yes | Yes | Yes | Yes | Yes | No | No | No | No |
| 6.97 | positive | No | Yes | No | Yes | No | No | Yes | Yes | No | Yes | No | No | Yes | No |
| 2.60 | negative | No | Yes | No | Yes | Yes | No | Yes | Yes | No | No | No | No | No | Yes |
| 4.08 | negative |  |  |  |  |  |  |  |  |  |  |  |  |  |  |
| 8.80 | positive | No | Yes |  | Yes |  | Yes | Yes | Yes | Yes | No | No | No | No | No |
| 6.61 | negative | No | Yes | No | No | No | No | Yes | Yes | No | No | No | No | No | Yes |
| 4.36 | positive | No | No | No | Yes | No | No | Yes | No | No | No | No | No | Yes | No |
| 4.18 | negative | No | No | No | No | Yes | Yes | Yes | Yes | Yes | Yes | No | No | Yes | Yes |
| 5.38 | positive |  |  |  |  |  |  |  |  |  |  |  |  |  |  |
| 6.04 | positive | No | No | No | No | No | No | No | No | Yes | No | No | No | No | No |
| 7.08 | positive | No | Yes | No | No | No | No | No | No | No | No | No | No | No | No |
| 7.45 | positive | Yes | Yes | Yes | No | No | No | Yes | Yes | No | No | No | No | No | No |
| 4.26 | positive | No | Yes | Yes | No | No | Yes | Yes | Yes | No | No | No | Yes | Yes | No |
| 7.79 | positive | No | Yes | Yes | No | No | No | No | Yes | No | Yes | No | No | No | No |
| 6.07 | negative | No | No | No | No | No | No | Yes | No | No | No | No | No | Yes | No |
| 8.25 | positive | No | Yes | Yes | No | No | Yes |  | Yes | Yes | No | No | No | No | No |
| 7.07 | positive | No | Yes | No | No | No | Yes | Yes | No | Yes | No | No | Yes | No | No |
| 9.34 | positive | No | Yes | Yes | No | No | Yes | No | No | Yes | No | No | No | No | No |
| 9.08 | positive | No | Yes | No | No | No | No | Yes | No | Yes | No | Yes | No | No | No |
| 7.28 | positive | No | No | No | Yes | No | Yes | Yes | Yes | Yes | No | No | No | Yes | No |
| 7.53 | positive | No | Yes | No | Yes | No | Yes | Yes | Yes | Yes | No | No | Yes |  | No |
| 5.47 | positive | No | Yes | No | Yes | No | No | No | No | Yes | No | No | No | No | No |
| 9.03 | positive | Yes | No | No | No | No | No | Yes | No | No | No | No |  | No | No |
| 8.99 | positive | Yes | Yes | No | No | No | Yes | Yes | No | Yes | No | No | No | No | No |
| 8.61 | positive | No | Yes | No | Yes | No | Yes | No | No | No | No | No | No | No | No |
| 8.12 | positive | No | No | No | Yes | Yes | No | No | Yes | No | No | No | No | Yes | No |
| 9.37 | positive | No | Yes | No | No | No | No | No | Yes | Yes | No | No | No | No | Yes |
| 6.58 | positive | No | Yes | No | Yes | No | Yes | Yes | No | No | No | No | No | Yes | No |
| 6.19 | positive | No | No | No | No | No | Yes | Yes | Yes | Yes | Yes | No | Yes | Yes | No |
| 6.93 | positive | No | No | No | No | No | No | Yes | No | Yes | No | No | No | Yes | No |
| 4.94 | negative | No | No | No | Yes | No | No | No | No | No | No | No | No | No | No |
| 9.43 | positive | Yes | Yes | No | Yes | No | Yes | Yes | Yes | No | No | No | No | No | No |
| 5.04 | negative | No | Yes | No | No | No | No | Yes | No | No | No | No | No | Yes | No |
| 9.30 | negative | No | No | No | Yes | No | No | Yes | No | No | No | No | No | No | No |
| 8.77 | positive | Yes | Yes | No | No | Yes | Yes | Yes | No | Yes | No | No | No | No | No |
| 8.10 | positive | No | Yes | No | No | No | No | No | No | Yes | No | No | No | No | No |
| 9.29 | positive | No | Yes | No | No | No | No | Yes | No | No | No | No | No | No | No |
| 6.97 | negative | No | No | No | No | No | No | No | No | No | No | No | No | Yes | No |
| 4.94 | positive | No | No | No | No | No | No | Yes | No | Yes | No | No | No | Yes | No |
| 8.14 | positive | Yes | Yes | Yes | Yes | No | Yes | Yes | Yes | Yes | No | Yes | No | Yes | No |
| 5.61 | positive | Yes | Yes | No | No | No | No | Yes | No | No | No | No | No | No | No |
| 6.94 | positive | No | No | No | No | No | No | No | No | Yes | No | No | No | Yes | No |
| 7.07 | positive | No | No | No | No | No | No | No | No | No | No | No | No | Yes | No |
| 5.76 | positive | No | No | No | No | No | No | No | No | No | No | No | No | Yes | No |
| 5.68 | positive | No | No | No | No | No | No | Yes | No | No | No | No | No | Yes | No |
| 8.56 | positive | Yes | No |  | No | No | No | Yes | No | No | No | No | No | No | No |
| 5.42 | negative | No | No | No | No | No | No | No | No | No | No | No | No | Yes | No |
| 6.80 | positive | No | Yes | No | No | No | No | Yes | No | No | No | No | No | Yes | No |
| 5.95 | positive | No | No | No | No | No | Yes | Yes | No | Yes | No | No | No | Yes | No |
| 3.72 | negative | No | Yes | No | No | No | No | No | Yes | No | No | No | No | Yes | No |
| 3.57 | negative | No | No | No | No | No | No | No | No | No | No | No | No | Yes | No |
| 6.23 | negative | No | Yes | No | No | No | No | Yes | No | Yes | No | No | No | Yes | No |
| 6.87 | positive | No | No | No | No | No | No | No | Yes | No | No | No | No | Yes | No |
| 8.08 | negative | No | Yes | No | No | No | Yes | Yes | No | No | No | No | No | No | No |
| 6.95 | positive | No | No | No | Yes | No | No | No | No | No | No | No | No | No | No |
| 5.88 | positive | No | Yes | Yes | No | No | No | No | No | No | No | No | No | Yes | No |
| 6.81 | positive | No | No | No | No | No | No | No | No | Yes | No | No | No | No | No |
| 8.65 | positive | No | No | No | No | No | Yes | Yes | No | Yes | No | No | No | No | No |
| 6.83 | positive | No | No | No | Yes | No | No | Yes | Yes | No | No | No | No | Yes | No |
| 8.64 | positive | No | Yes | No | Yes | No | Yes | Yes | Yes | No | No | No | No | Yes | No |
| 7.85 | positive | No | Yes | Yes | Yes | No | Yes | Yes | Yes | Yes | No | Yes | No | No | No |
| 8.32 | positive | No | No | No | Yes | No | Yes | Yes | Yes | No | No | No |  | No | No |
| 9.27 | negative | No | Yes |  | Yes | No | No | Yes | Yes | No | No | No |  | No | No |
| 8.89 | positive | Yes | Yes | Yes | Yes | No | Yes | No | Yes | Yes | No | Yes | No | Yes | No |
| 4.46 | positive | Yes | No | No | Yes | No | No | Yes | Yes | No | No | Yes | No | Yes | No |
| 6.58 | positive | No | No | No | Yes | No | No | No | No | No | No | No | No | Yes | No |
| 7.75 | negative | No | Yes | No | No | No | Yes | Yes | Yes | Yes | No | No | No | No | No |
| 9.69 | positive | Yes | No | No | No | No | Yes | Yes | No | No | No | No | No | Yes |  |
| 8.91 | positive | No | Yes | No | Yes | No | Yes | Yes | Yes | No | No | Yes | No | Yes | No |
| 8.70 | positive | Yes | Yes | Yes | No | No | No | No | Yes | No | No | No | No | No | No |
| 8.17 | positive | No | Yes |  | No | No | No | Yes | No | No | No | No | No | No | No |
| 8.72 | positive | No | Yes | No | No | No | No | No | No | No | No | No | No | Yes | No |
| 3.83 | negative | No | No | No | Yes | No | No | No | No | No | No | No | No | No | No |
| 7.86 | positive | No | Yes | No | Yes | No | Yes | Yes | Yes | Yes | No | No | No | Yes | No |
| 8.75 | positive | No | No | No | No | No | Yes | No | No | No | No | No | No | No | No |
| 7.82 | positive | No | Yes | Yes | No | No | No | Yes | Yes | No | No | Yes | No | No | No |
| 8.01 | positive | No | No | No | Yes | No | No | No | No | Yes | No | No | No | Yes | No |
| 9.08 | positive | No | Yes | No | No | No | Yes | No | Yes | No | No | No | No | No | Yes |
| 9.63 | positive | No | No | No | No | No | No | Yes | No | Yes | No | No | No | No | No |
| 7.47 | positive | No | Yes | Yes | No | No | Yes | Yes | Yes | Yes | No | Yes | Yes | No | No |
| 7.24 | positive | No | No | No | No | No | Yes | No | Yes | Yes | No | No | No | Yes | No |
| 8.67 | positive | Yes | Yes | No | No | No | Yes | Yes | Yes | Yes | No | No | No | Yes | No |
| 8.47 | positive | Yes | No | Yes | No | No | Yes | Yes | Yes | No | No | No | No | No | No |
| 6.03 | negative | No | No | No | No | No | No | Yes | Yes | No | No | No | No | Yes | No |
| 5.86 | negative | Yes | No | No | No | No | Yes | Yes | Yes | No | No | No | No | Yes | No |
| 6.81 | positive | No | Yes | Yes | Yes | No | Yes | Yes | Yes | Yes | No | No | No | Yes | No |
| 4.70 | negative | No | Yes | No | No | No | No | No | No | No | No | No |  | Yes |  |
| 6.61 | positive | No | Yes |  | No | No | No | No | No | Yes | No | No |  | Yes | No |
| 7.34 | positive |  |  |  |  |  | Yes |  |  |  |  |  |  | Yes |  |
| 8.26 | positive | No | Yes | Yes | Yes | No | No | Yes | No | No | No | No | No | Yes | No |
| 5.61 | positive | No | Yes | No | No | No | No | No | Yes | No | No | No | No | Yes | No |
| 9.00 | positive | Yes | No | No | No | No | No | Yes | No | Yes | No | No | No | Yes | No |
| 8.38 | positive | Yes | No | No | No | No | No | No | No | No | No | No | No | No | Yes |
| 9.19 | positive | No | No | No | Yes | No | Yes | Yes | Yes | No | Yes | No | No | Yes | No |

# **(G) Table 3: Antigen-based RDT with test result, CT values and viral load for PCR positive participants in Berlin and Heidelberg**

CT-value reported here represent the E-gene genome target (similar to the target described by Corman) in descending order. A conversion of CT-values for RT-PCR tests into viral-load was performed using quantified specific in vitro-transcribed RNA (Corman 2020 Eurosurveillance).

| **Berlin** | | | |
| --- | --- | --- | --- |
| **Antigen RDT result** | **CT-value**  (E-Gene) | **Viral load**  (log_10_ SARS-CoV2 RNA copies /mL) | **PCR assay** |
| positive | 14.61 | 9.63 | TIB Molbiol |
| positive | 15.29 | 9.43 | TIB Molbiol |
| positive | 15.51 | 9.37 | TIB Molbiol |
| positive | 15.60 | 9.34 | TIB Molbiol |
| negative | 15.74 | 9.30 | TIB Molbiol |
| positive | 15.76 | 9.29 | TIB Molbiol |
| negative | 15.84 | 9.27 | TIB Molbiol |
| positive | 16.09 | 9.19 | TIB Molbiol |
| positive | 16.46 | 9.08 | TIB Molbiol |
| positive | 16.64 | 9.03 | TIB Molbiol |
| positive | 16.76 | 9.00 | TIB Molbiol |
| positive | 17.12 | 8.89 | TIB Molbiol |
| positive | 17.53 | 8.77 | TIB Molbiol |
| positive | 17.60 | 8.75 | TIB Molbiol |
| positive | 17.69 | 8.72 | TIB Molbiol |
| positive | 17.75 | 8.70 | TIB Molbiol |
| positive | 17.85 | 8.67 | TIB Molbiol |
| positive | 17.93 | 8.65 | TIB Molbiol |
| positive | 18.04 | 8.61 | TIB Molbiol |
| positive | 18.82 | 8.38 | TIB Molbiol |
| positive | 19.04 | 8.32 | TIB Molbiol |
| positive | 19.23 | 8.26 | TIB Molbiol |
| positive | 19.27 | 8.25 | TIB Molbiol |
| positive | 19.63 | 8.14 | TIB Molbiol |
| positive | 19.70 | 8.12 | TIB Molbiol |
| positive | 19.77 | 8.10 | TIB Molbiol |
| negative | 19.85 | 8.08 | TIB Molbiol |
| positive | 20.08 | 8.01 | TIB Molbiol |
| positive | 20.58 | 7.86 | TIB Molbiol |
| positive | 21.88 | 7.47 | TIB Molbiol |
| positive | 22.34 | 7.34 | TIB Molbiol |
| positive | 22.52 | 7.28 | TIB Molbiol |
| positive | 22.66 | 7.24 | TIB Molbiol |
| positive | 23.25 | 7.07 | TIB Molbiol |
| negative | 23.57 | 6.97 | TIB Molbiol |
| positive | 23.65 | 6.95 | TIB Molbiol |
| positive | 23.69 | 6.94 | TIB Molbiol |
| positive | 23.91 | 6.87 | TIB Molbiol |
| positive | 24.10 | 6.81 | TIB Molbiol |
| positive | 24.11 | 6.81 | TIB Molbiol |
| positive | 24.15 | 6.80 | TIB Molbiol |
| positive | 24.78 | 6.61 | TIB Molbiol |
| negative | 26.07 | 6.23 | TIB Molbiol |
| negative | 26.61 | 6.07 | TIB Molbiol |
| positive | 26.99 | 5.95 | TIB Molbiol |
| positive | 27.23 | 5.88 | TIB Molbiol |
| negative | 27.30 | 5.86 | TIB Molbiol |
| positive | 28.14 | 5.61 | TIB Molbiol |
| positive | 28.16 | 5.61 | TIB Molbiol |
| positive | 28.62 | 5.47 | TIB Molbiol |
| negative | 28.78 | 5.42 | TIB Molbiol |
| negative | 31.20 | 4.70 | TIB Molbiol |
| negative | 34.14 | 3.83 | TIB Molbiol |
| negative | 34.50 | 3.72 | TIB Molbiol |
| negative | 35.00 | 3.57 | TIB Molbiol |

| **Berlin** | | | |
| --- | --- | --- | --- |
| **Antigen RDT result** | **CT-value**  (E-Gene) | **Viral load**  (log_10_ SARS-CoV2 RNA copies /mL) | **PCR assay** |
| positive | 17.47 | 9.69 | Roche Cobas |
| positive | 19.54 | 9.08 | Roche Cobas |
| positive | 19.85 | 8.99 | Roche Cobas |
| positive | 20.13 | 8.91 | Roche Cobas |
| positive | 21.04 | 8.64 | Roche Cobas |
| positive | 21.29 | 8.56 | Roche Cobas |
| positive | 21.60 | 8.47 | Roche Cobas |
| positive | 22.63 | 8.17 | Roche Cobas |
| positive | 23.7 | 7.85 | Roche Cobas |
| positive | 23.79 | 7.82 | Roche Cobas |
| positive | 23.89 | 7.79 | Roche Cobas |
| negative | 24.05 | 7.75 | Roche Cobas |
| positive | 24.79 | 7.53 | Roche Cobas |
| positive | 26.34 | 7.07 | Roche Cobas |
| positive | 26.81 | 6.93 | Roche Cobas |
| positive | 27.15 | 6.83 | Roche Cobas |
| positive | 27.98 | 6.58 | Roche Cobas |
| positive | 27.99 | 6.58 | Roche Cobas |
| positive | 29.32 | 6.19 | Roche Cobas |
| negative | 29.87 | 6.03 | Roche Cobas |
| positive | 30.75 | 5.76 | Roche Cobas |
| positive | 31.05 | 5.68 | Roche Cobas |
| negative | 33.21 | 5.04 | Roche Cobas |
| positive | 33.53 | 4.94 | Roche Cobas |
| negative | 33.55 | 4.94 | Roche Cobas |
| positive | 35.15 | 4.46 | Roche Cobas |

| **Heidelberg** | | | |
| --- | --- | --- | --- |
| **Antigen RDT result** | **CT-value**  (E-Gene) | **Viral load**  (log_10_ SARS-CoV2 RNA copies /mL) | **PCR assay** |
| positive | 16.77 | 8.80 | Seegene |
| positive | 17.50 | 8.59 | Seegene |
| positive | 17.88 | 8.48 | Seegene |
| positive | 17.93 | 8.48 | Seegene |
| positive | 18.22 | 8.38 | Seegene |
| positive | 18.42 | 8.32 | Seegene |
| positive | 18.80 | 8.20 | Seegene |
| positive | 18.95 | 8.15 | Seegene |
| positive | 19.12 | 8.11 | Seegene |
| positive | 19.15 | 8.08 | Seegene |
| positive | 19.27 | 8.08 | Seegene |
| positive | 19.45 | 8.00 | Seegene |
| positive | 19.52 | 8.00 | Seegene |
| positive | 19.60 | 7.97 | Seegene |
| positive | 20.02 | 7.85 | Seegene |
| positive | 20.05 | 7.83 | Seegene |
| positive | 20.16 | 7.79 | Seegene |
| positive | 20.27 | 7.76 | Seegene |
| positive | 20.41 | 7.73 | Seegene |
| positive | 20.43 | 7.73 | Seegene |
| positive | 20.50 | 7.71 | Seegene |
| positive | 20.80 | 7.61 | Seegene |
| positive | 20.84 | 7.61 | Seegene |
| positive | 20.95 | 7.56 | Seegene |
| positive | 20.99 | 7.56 | Seegene |
| positive | 21.13 | 7.53 | Seegene |
| positive | 21.36 | 7.45 | Seegene |
| positive | 21.54 | 7.41 | Seegene |
| positive | 22.42 | 7.15 | Seegene |
| negative | 22.47 | 7.11 | Seegene |
| positive | 22.55 | 7.08 | Seegene |
| positive | 22.76 | 7.04 | Seegene |
| positive | 23.04 | 6.97 | Seegene |
| positive | 23.07 | 6.94 | Seegene |
| positive | 23.40 | 6.85 | Seegene |
| positive | 23.47 | 6.82 | Seegene |
| positive | 23.52 | 6.82 | Seegene |
| positive | 24.04 | 6.67 | Seegene |
| negative | 24.21 | 6.61 | Seegene |
| positive | 24.30 | 6.58 | Seegene |
| positive | 25.23 | 6.32 | Seegene |
| positive | 25.33 | 6.28 | Seegene |
| positive | 25.70 | 6.18 | Seegene |
| positive | 26.08 | 6.04 | Seegene |
| positive | 26.13 | 6.04 | Seegene |
| positive | 26.34 | 5.99 | Seegene |
| positive | 27.81 | 5.56 | Seegene |
| positive | 27.9 | 5.52 | Seegene |
| positive | 28.28 | 5.40 | Seegene |
| positive | 28.38 | 5.38 | Seegene |
| positive | 29.30 | 5.11 | Seegene |
| positive | 30.02 | 4.90 | Seegene |
| negative | 30.25 | 4.81 | Seegene |
| positive | 30.40 | 4.79 | Seegene |
| positive | 31.82 | 4.36 | Seegene |
| negative | 32.18 | 4.26 | Seegene |
| positive | 32.19 | 4.26 | Seegene |
| negative | 32.46 | 4.18 | Seegene |
| negative | 32.75 | 4.08 | Seegene |
| positive | 33.05 | 3.99 | Seegene |
| positive | 33.13 | 3.99 | Seegene |
| negative | 33.83 | 3.78 | Seegene |
| negative | 34.16 | 3.66 | Seegene |
| negative | 36.61 | 2.95 | Seegene |
| negative | 37.82 | 2.60 | Seegene |

# **(H) Figure 2: Correlation between the cut-off-index value and the viral load for all RT-PCR positive cases**


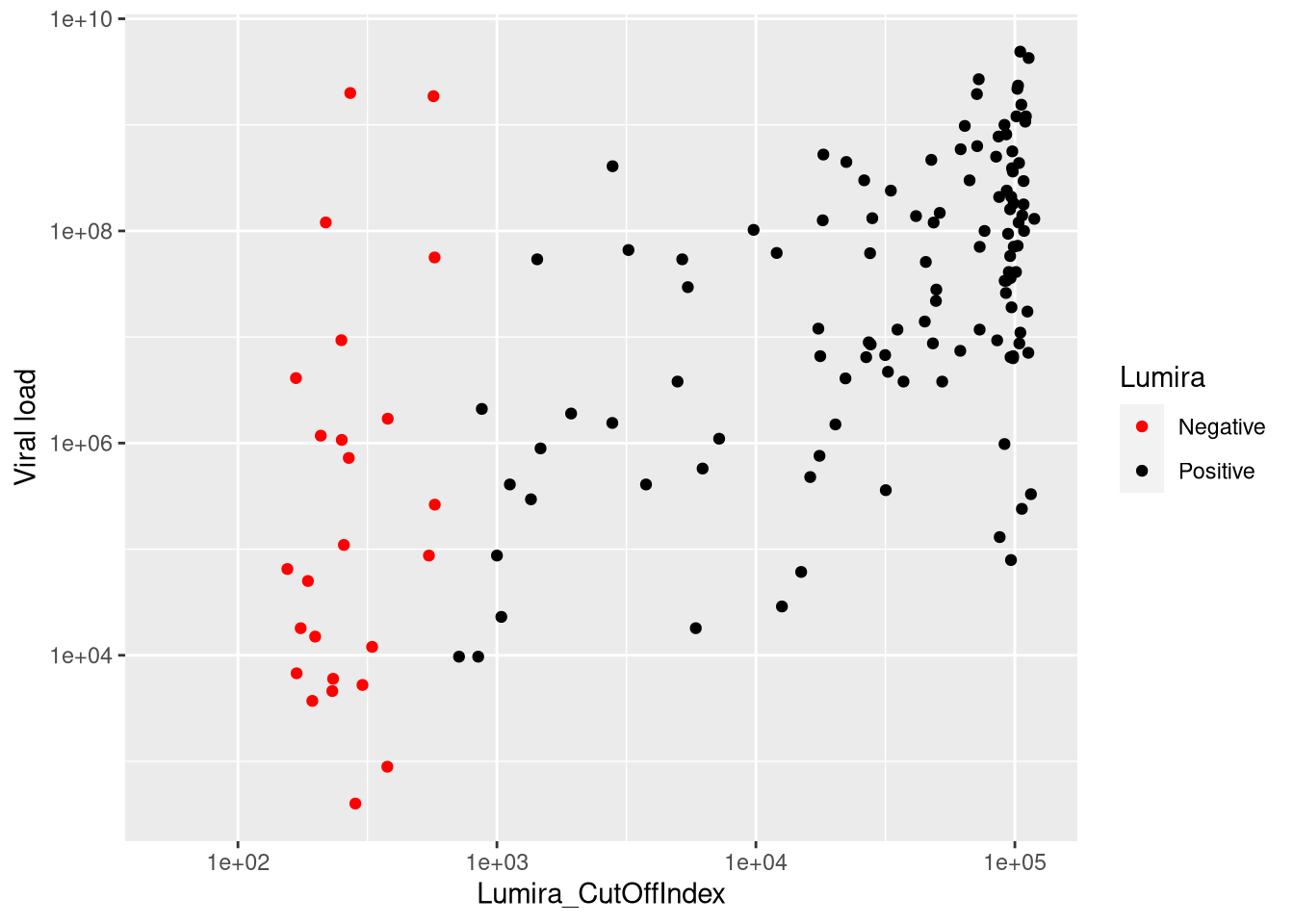


# **(I) Table 4: Discrepant analysis**

| **Study ID** | **PCR** | **Ag-Test Lumira** | **COI** | **CT value: E-Gene** | **Viral load** | **Buffer PCR** | **Buffer CT value: E-Gene** | **Buffer Viral load** |
| --- | --- | --- | --- | --- | --- | --- | --- | --- |
| **False Negatives** |  |  |  |  |  |  |  |  |
| **Heidelberg** |  |  |  |  |  |  |  |  |
| 4300 | positive | negative |  | 22,47 | 7,11 |  |  |  |
| 4398 | positive | negative |  | 32,18 | 4,26 |  |  |  |
| 4433 | positive | negative |  | 33,83 | 3,78 |  |  |  |
| 4530 | positive | negative |  | 36,61 | 2,95 | negative |  |  |
| 4578 | positive | negative |  | 30,25 | 4,81 | negative |  |  |
| 4678 | positive | negative |  | 34,16 | 3,66 | negative |  |  |
| 4696 | positive | negative | 284 | 37,82 | 2,6 | negative |  |  |
| 4719 | positive | negative | 330 | 32,75 | 4,08 | negative |  |  |
| 4722 | positive | negative | 168 | 24,21 | 6,61 | negative |  |  |
| 4726 | positive | negative | 199 | 32,46 | 4,18 | negative |  |  |
| **Berlin** |  |  |  |  |  |  |  |  |
| A849 | positive | negative |  | 26,61 | 6,07 |  |  |  |
| B974 | positive | negative | 546 | 33,55 | 4,94 |  |  |  |
| B981 | positive | negative |  | 33,21 | 5,04 |  |  |  |
| B984 | positive | negative |  | 15,74 | 9,3 | positive | 34,9 | 4,52 |
| B994 | positive | negative | 251 | 23,57 | 6,97 |  |  |  |
| B1017 | positive | negative | 575 | 28,78 | 5,42 |  |  |  |
| B1029 | positive | negative | 303 | 34,5 | 3,72 |  |  |  |
| B1030 | positive | negative | 194 | 35 | 3,57 |  |  |  |
| B1032 | positive | negative | 379 | 26,07 | 6,23 |  |  |  |
| B1048 | positive | negative | 218 | 19,85 | 8,08 |  |  |  |
| V1032 | positive | negative |  | 15,84 | 9,27 | positive |  | < 100 CP/mL |
| V1042 | positive | negative |  | 24,05 | 7,75 | positive | 37,82 | 2,61 |
| V1063 | positive | negative | 168 | 34,14 | 3,83 |  |  |  |
| V1276 | positive | negative | 252 | 29,87 | 6,03 |  |  |  |
| V1278 | positive | negative | 268 | 27,03 | 5,86 |  |  |  |
| V1280 | positive | negative | 186 | 31,2 | 4,7 |  |  |  |
| **False Positives** |  |  |  |  |  |  |  |  |
| **Heidelberg** |  |  |  |  |  |  |  |  |
| 4483 | negative | positive |  |  |  | negative |  |  |
| 4568 | negative | positive |  |  |  | negative |  |  |
| 4580 | negative | positive |  |  |  | negative |  |  |
| **Berlin** |  |  |  |  |  |  |  |  |
| V1092 | negative | positive | 687 |  |  |  |  |  |

# **(J) Figure 3: Specific infectivity of virus stocks**

**
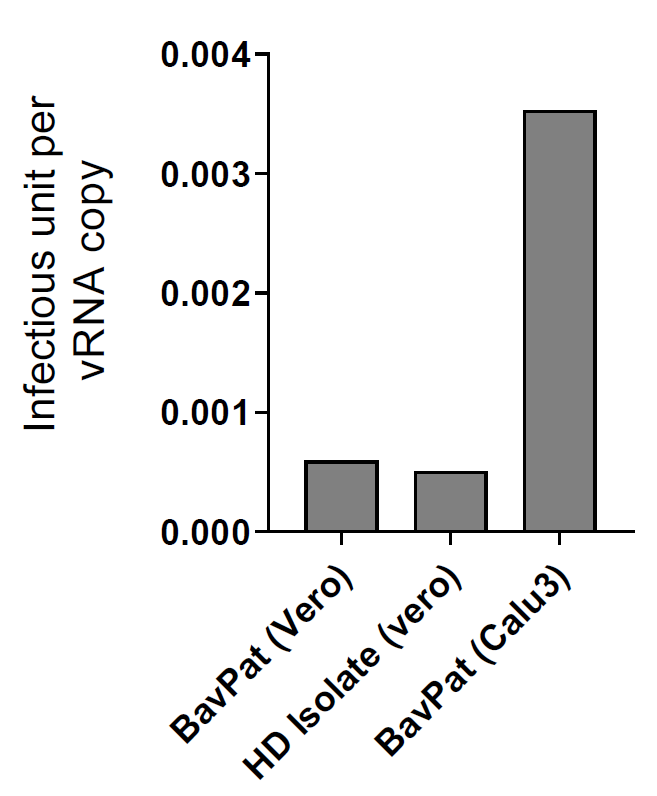
**

For RNA copy number quantification, RNA was isolated for 50 µl of virus stock with the Nucleospin RNA extraction kit (Macherey-Nagel) according to the manufacturer´s instructions. To create standard for absolute quantification, SARS-CoV-2 NC sequence was reverse transcribed from total RNA isolated from cells infected with the SARS-CoV-2 BavPat1 stain using Superscript 3 and specific primers (TTAGGCCTGAGTTGAGTCA). The resulting cDNA was amplified and cloned into the pJET1.2 plasmid. Ten microgram of plasmid DNA was linearized by AdeI restriction enzyme digestion and DNA was purified using the NucleoSpin Gel and PCR Clean-up kit (Macherey-Nagel: 740609.250). For *in vitro* transcription, T7 RNA polymerase was used as previously described (Fischl and Bartenschlager, 2013). *In vitro* transcripts were purified by phenol-chloroform extraction and resuspended in RNase-free water. RNA integrity was confirmed by agarose gel electrophoresis. TaqMan RT-qPCR was done using the QuantaBio Script XLT One-Step RT-qPCR. The primers and probe used were: SARS-CoV-2-N (forward) 5’-TTA CAA ACA TTG GCC GCA AA-3’, SARS-CoV-2-N (reverse) 5’-GCG CGA CAT TCC GAA GAA-3’, SARS-CoV-2-N (probe) 5’-FAM-ACA ATT TGC CCC CAG CGC TTC AG-BHQ1-3’. Absolute quantification was done by comparing to a standard curve from known quantities of the *in vitro* transcribed NC gene RNA.

Figure 3: Quantification of virus stock specific infectivity. Virus titers for each stock were determined by plaque assay. Viral RNA (vRNA) copy number per milliliter was calculated by absolute RT-qPCR using *in vitro* transcribed NC gene RNA as a standard. The graph shows the specific infectivity (infectious unit per viral RNA copy) for each virus stock.

# **(K) Table 5: Exclusivity testing of LumiraDx™**

| Pathogen | CT (lowest - highest value)  *concentration (colony forming units [CFU] /ml)* | LumiraDx™ | Cross-Reactivity |
| --- | --- | --- | --- |
| Coronavirus OC 43 Coronavirus NL 63 Coronavirus 229 E Coronavirus HKU 1 | 19.8 - 27.7  25.9 - 30.6  22.7 - 27.0  18.1 - 31.5 | 3/3 negative 3/3 negative 3/3 negative 3/3 negative | no |
| Adenovirus | 26.7 - 35 | 5/5 negative | no |
| Bocavirus | 31.5 - 33.2 | 2/2 negative | no |
| Influenza A H3N2 virus Influenza A H1N1 virus Influenza B virus | 17.6 - 30.7  19.4 - 29.9  16.8 - 30.5 | 4/4 negative 3/3 negative 3/3 negative | no |
| Metapneumovirus | 26.2 - 32.7 | 4/4 negative | no |
| Parainfluenza virus | 17.4 - 32.5 | 10/10 negative | no |
| Respiratory syncytial virus | 16.4 - 31.2 | 10/10 negative | no |
| Rhinovirus | 28.5 - 35 | 10/10 negative | no |
| *Mycoplasma pneumoniae* | 23.2 - 35 | 8/8 negative | no |
| *Staphylococcus aureus* | Unknown (clinical samples) | 9/9 negative | no |
| *Streptococcus sp*. | 1x10^7^ CFU/ml | 7/7 negative | no |

**(L) Figure 4: System Usability Score and Ease-of-Use assessment results**


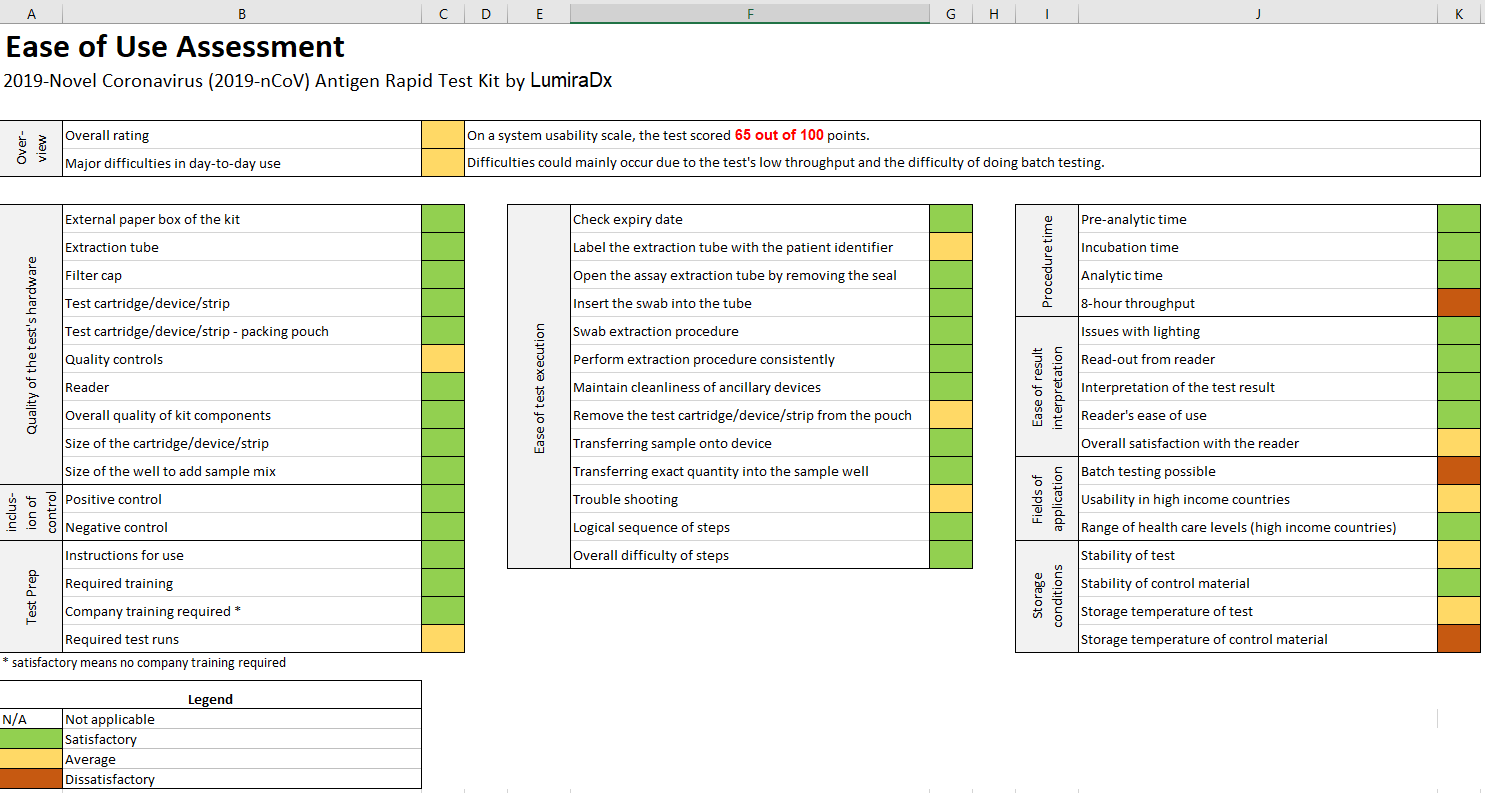


# **(M) Table 6: Comorbidities and list of symptoms of participants overall, Berlin and Heidelberg**

|  | **Overall** | **Heidelberg** | **Berlin** |
| --- | --- | --- | --- |
| **Data combined: Overweight and Adipositas > BMI 25** – Information available on N=703 | | | |
| Yes | 327  (46.5%) | 243  (50.2%) | 84  (38.4%) |
| No | 376  (53.5%) | 241  (49.8%) | 135  (61.6%) |
| **Comorbidities** – Information available on N=760 | | | |
| All with comorbidities | 241 | 183 | 58 |
| Lung diseases | | | |
| Asthma bronchiale | 60 | 39 | 21 |
| Chronic obstructive pulmonary disease (COPD) | 7 | 5 | 2 |
| Breathing disorders during sleep and obstructive Sleep Apnea  (OSAS) | 9 | 9 | 0 |
| Interstitial Lung Disease | 0 | 0 | 0 |
| Lung Cancer | 2 | 1 | 1 |
| Other | 13 | 11 | 2 |
| Other diseases | | | |
| Cardiovascular diseases | 80 | 63 | 17 |
| Chronic kidney diseases | 9 | 8 | 1 |
| Autoimmune | 51 | 48 | 3 |
| HIV | 1 | 1 | 0 |
| Others | 79 | 52 | 27 |
| List of symptoms reported | | | |
| Fever | 90  (19.0%) | 49  (23.3%) | 41  (15.5%) |
| Fever measured   1. <38.4 2. >38.5 and <=39.4 3. >39.5 and >40.5 | 52(74.3%)  17(24.3%)  1 (1.4%) | 37(82.2%)  8(17.8%)  0 | 15(60%)  9(36%)  1(4%) |
| Cough | 247  (51.6%) | 112  (52.6%) | 135  (50.8%) |
| Productive cough | 72 (15.5%) | 40 (18.9%) | 32 (12.6%) |
| Sore throat | 242  (50.2%) | 115  (53.5%) | 127  (47.6%) |
| Shortness of breath | 46  (9.7%) | 31  (14.8%) | 15  (5.7%) |
| Muscle pain | 176  (36.7%) | 81  (38.2%) | 95  (35.4%) |
| Fatigue | 297  (62.0%) | 143  (66.8%) | 154  (58.1%) |
| Headache | 251  (52.3%) | 130  (60.7%) | 121  (45.5%) |
| Runny nose | 197  (41.5%) | 87  (41.6%) | 110  (41.4%) |
| Chest pain | 41  (8.6%) | 35  (16.6%) | 6  (2.3%) |
| Diarrhea | 54  (11.3%) | 25  (11.8%) | 29  (10.8%) |
| Nausea | 25  (5.4%) | 16  (4.3%) | 9  (6.3%) |
| Loss of taste and smell | 104  (21.8%) | 32  (15.3%) | 72  (27.0%) |

# **(N) Table 7: Sensitivity and Specificity overall and by subgroups**

|  | **Overall**  N | **Ag-Test positive/ PCR positive**  N  (%) | **Ag-Test negative/ PCR positive**  N  (%) | **Ag-Test positive/ PCR negative**  N  (%) | **Ag-Test negative/ PCR negative**  N  (%) | **Sensitivity**  %  (95% CI) | **Specificity**  %  (95% CI) |
| --- | --- | --- | --- | --- | --- | --- | --- |
| **Sensitivity** | | | | | | | |
| **Overall** | 761 | 120  (15.8) | 26  (3.4) | 4  (0.5) | 611  (79.7) | 82.2  (75.2-87.5) | 99.3  (98.3-99.7) |
| **Heidelberg** | 488 | 55  (11.3) | 10  (2.0) | 3  (0.6) | 420  (86.1) | 84.6  (73.9-91.4) | 99.3  (97.9-99.8) |
| **Berlin** | 273 | 65  (23.8) | 16  (5.9) | 1  (0.4) | 191  (70.0) | 80.2  (70.3-87.5) | 99.5  (97.1-100) |
| **Symptom duration** – Information available for N=472 | | | | | | | |
| **0-7 days**  Overall | 423 | 102  (24.1) | 16  (3.8) | 2  (0.5) | 303  (71.6) | 86.4  (79.1-91.5) | 99.3  (97.6-99.8) |
| **8-14 days**  Overall | 39 | 7  (17.9) | 6  (15.4) | 0 | 26  (66.7) | 53.8  (29.1-76.8) | 100  (87.1-100) |
| **Symptomatic versus Asymptomatic** – Information available for N= 472 | | | | | | | |
| **Symptomatic** | 210 | 45  (21.4) | 8  (3.8) | 2  (1.0) | 155  (73.8) | 84.9  (72.9-92.1) | 98.7  (95.5-99.6) |
| **Asymptomatic** | 271 | 7  (2.6) | 2  (0.7) | 1  (0.4) | 261  (96.3) | 77.8  (45.3-93.7) | 99.6  (97.9-100) |
| **CT-Value PCR <30 and >=30** – Information available for N=146 | | | | | | | |
| **CT value PCR <30** | 122 | 110 | 12 | NA | NA | 90.2  (83.6-94.3) | NA |
| **CT value PCR ≥30** | 24 | 10 | 14 | NA | NA | 41.7  (24.5-61.2) | NA |
| **CT-Value PCR <25 and >=25** – Information available for N=146 | | | | | | | |
| **CT value PCR <25** | 95 | 88 | 7 | NA | NA | 92.6  (85.6-96.4) | NA |
| **CT value PCR ≥25** | 51 | 32 | 19 | NA | NA | 62.7  (49.0-74.7) | NA |
